# Objective and subjective voice outcomes after total laryngectomy: a systematic review

**DOI:** 10.1007/s00405-017-4790-6

**Published:** 2017-10-31

**Authors:** Klaske E. van Sluis, Lisette van der Molen, Rob J. J. H. van Son, Frans J. M. Hilgers, Patrick A. Bhairosing, Michiel W. M. van den Brekel

**Affiliations:** 1 Department of Head and Neck Oncology and Surgery, Netherlands Cancer Institute-Antoni van Leeuwenhoek, Plesmanlaan 121, 1066 CX Amsterdam, The Netherlands; 2 Amsterdam Center for Language and Communication, University of Amsterdam, Amsterdam, The Netherlands; 3 Department of Maxillofacial Surgery, Academic Medical Center, Amsterdam, The Netherlands; 4 Scientific Information Service, The Netherlands Cancer Institute-Antoni van Leeuwenhoek, Amsterdam, The Netherlands

**Keywords:** Total laryngectomy, Voice, Speech, Acoustic, Perceptual, Self-evaluation

## Abstract

**Background:**

Esophageal speech (ES), tracheoesophageal speech (TES) and/or electrolarynx speech (ELS) are three speech rehabilitation methods which are commonly provided after total laryngectomy (TL).

**Methods:**

A systematic review of the literature was conducted to evaluate comparative acoustic, perceptual, and patient-reported outcomes for ES, TES, ELS and healthy speakers.

**Results:**

Twenty-six articles could be included. In most studies, methodological quality was low. It is likely that an inclusion bias exists, many studies only included exceptional speakers. Significant better outcomes are reported for TES compared to ES for the acoustic parameters, fundamental frequency, maximum phonation time and intensity. Perceptually, TES is rated with a significant better voice quality and intelligibility than ES and ELS. None of the speech rehabilitation groups reported clearly better outcomes in patient-reported outcomes.

**Conclusions:**

Studies on speech outcomes after TL are flawed in design and represent weak levels of evidence. There is an urge for standardized measurement tools for evaluations of substitute voice speakers. TES is the favorable speech rehabilitation method according to acoustic and perceptual outcomes. All speaker groups after TL report a degree of voice handicap. Knowledge of caretakers and differences in health care and insurance systems play a role in the speech rehabilitation options that can be offered.

**Electronic supplementary material:**

The online version of this article (doi:10.1007/s00405-017-4790-6) contains supplementary material, which is available to authorized users.

## Introduction

As a consequence of total laryngectomy (TL), patients lose their natural voice, making speech rehabilitation with a substitute sound source a major rehabilitation goal. The three main rehabilitation options are esophageal speech (ES), tracheoesophageal speech (TES), and electrolarynx speech (ELS) [1]. ES and TES have in common that the substitute sound source is internal, i.e., the voice is produced in the pharyngoesophageal (PE) segment. ES is performed by administering air into the esophagus, which is subsequently expelled, causing mucosal vibrations in the PE-segment. In TES, pulmonary air channeled through a voice prosthesis or tracheoesophageal (TE) fistula. The voice prosthesis enables pulmonary air to enter the esophagus, and prevents esophageal content from entering the airway. In TES, pulmonary air is the driving force for the mucosal vibrations in the PE-segment. In ELS the substitute sound source is external: an electrolarynx is a sound producing, mostly handheld device, which can be placed against the neck or cheek [1].

Worldwide, no evidence-based consensus exists on which speech rehabilitation method is best for restoring oral communication. It is often assumed that for TL patients a better voice quality is associated with an improved quality of life [2, 3].

For evaluating speech rehabilitation outcomes, multidimensional assessment is recommended [4, 5]. This systematic review focuses on acoustic analysis, perceptual evaluation, and patient-reported outcomes (PROs) of the three substitute speech options. Acoustic voice analysis regularly includes pitch and amplitude measurements [6]. However, standard acoustic voice analyses are not always sufficient to measure substitute voices, because speech originating in the vibrating PE-segment, ES and TES, is known to contain more noise components and less regularity than laryngeal voice [7]. Perceptual evaluations of the speech rehabilitation methods also require a well-thought-out approach because of the deviances in regularity compared to laryngeal voices [8, 9]. Most convenient for such evaluations of substitute voices are overall impression of voice quality and impression of speech intelligibility [8, 9]. Results of speech rehabilitation from a patient’s perspective are mostly evaluated by Quality of Life (QOL) questionnaires such as those of the European Organization for Research and Treatment of Cancer, Quality of Life Questionnaire (EORTC), the module for patients with head and neck cancer 35-item version (EORTC QLQ-H&N35) and/or the EORTC QLQ-C30 questionnaire, which include questions about speech functioning [10, 11]. PROs, such as the Voice Handicap Index (VHI) and Voice-Related Quality of Life (V-RQOL) provide more detailed evaluations of speech rehabilitation results [12–14].

At present, a comprehensive literature review on the advantages and disadvantages of the current speech rehabilitation options has not been performed. Collecting the best evidence available on the three speech rehabilitation methods will likely help to build consensus about which speech rehabilitation after TL is optimal, and could aid in clinicians’ decision-making, patients’ counseling and reimbursement issues. In this systematic review, we focus on obtaining comparative acoustic, perceptual, and PROs for the three speech rehabilitation methods after TL. We aim to identify how outcomes of the various speech rehabilitation methods relate to those of normal laryngeal speech (healthy speakers), and what outcomes are favorable for each rehabilitation method.

## Materials and methods

The literature on speech outcomes after total laryngectomy (TL) was reviewed by means of a systematic search strategy. This search strategy was conducted with specific attention to the primary and secondary outcomes of interest (Table 1). The most suitable primary and secondary outcomes were selected based on the literature. With the acoustic outcomes, we aimed to obtain objective information about the speech rehabilitation options. We aimed to obtain subjective information of the voices though perceptual ratings and PROs. We have chosen to indicate fundamental frequency (*F*_0_), Harmonics to Noise Ratio (HNR), and percentage of voicedness (%voiced) as primary acoustic outcomes. These outcomes are indicated by several authors to obtain information about the pitch, stability and the amount of noise components [7, 15–17]. Secondary acoustic outcomes of interest were jitter, shimmer, intensity, spectral tilt and maximum phonation time (MPT). These outcome variables are frequently used in the literature although some are known to be less reliable in substitute voicing [16, 17]. Primary perceptual outcomes of interest were overall impression of voice quality and intelligibility, derived from the IINFVo scale, where impression, intelligibility, noise, fluency, and voicing is evaluated [18]. Secondary perceptual outcomes of interest were chosen from well-established perceptual assessment tools, such as the Grade Roughness Breathiness Asthenia Strain scale assessment (GRBAS [19]), and other recommended perceptual parameters in TL-speech such as unintended additive noise, fluency, and voicing [8, 18]. Primary PROs were the widely used VHI [13] and V-RQOL [14]. As secondary PROs we included voice specific outcomes on the EORTC QLQ-H&N35 [11] and the EORTC QLQ-C30 [10], where general quality of life is evaluated including a specific subset of questions on communication.

**Table 1:** Research questions and outcomes of interest

Research questions	Which comparative acoustic, perceptual, and PROs are available for TES, ES and ELS after TL? How do outcomes of TES, ES and ELS relate to those of normal laryngeal speech? What outcomes are favorable in which rehabilitation method?
Primary outcomes	
Acoustic	***F***_**0**_: fundamental frequency, a result of the rate of vibration of the (neo) glottis [15] **HNR**: harmonics to noise ratio, ratio between the total energy of the periodic voice signal and the energy of noise components [15, 17, 20] **%voiced**: the percentage voicedness [9, 16, 17]
Perceptual	**Voice quality**: impression of the overall voice quality [8, 9, 14] **Intelligibility**: impression of the intelligibility [8, 18]
PROs	**VHI**: Voice Handicap Index [13] **V-RQOL**: voice-related quality of life [14]
Secondary outcomes	
Acoustic	**Jitter**: relative variability in the period-to-period frequency [20, 21] **Shimmer**: relative variability in the peak-to-peak amplitude [20, 21] **Intensity**: Loudness dB [8, 22] **Spectral tilt**: a comparison between low frequency energy (between 0 and 1 kHz) and high frequency energy (between 1 and 5 kHz) [21, 23] **MPT**: Maximum Phonation Time [22, 24]
Perceptual	GRBAS: Grade Roughness Breathiness Asthenia Strain scale assessment [18, 19] **Unintended additive noise**: uncontrolled noises during speech [18] **Fluency**: the perceived smoothness of the sound production [18] **Voicing**: voicing is voiced or unvoiced where it is supposed to be voiced or unvoiced [18]
PROs	**EORTC QLQ-H&N35**: European Organization for Research and Treatment of Cancer, Quality of Life Questionnaire module for patients with head and neck cancer 35-item version [11] **EORTC QLQ-C30**: European Organization for Research and Treatment of Cancer, Quality of Life Questionnaire C30 [10]

*ES* esophageal speakers, *TES* tracheoesophageal speakers, *ELS* electrolarynx speakers, *PROs* patient-reported outcomes

The literature search was performed by the medical information specialist. The search was conducted in PubMed, Embase (ovid), Scopus and PsychInfo. Terms searched for were “laryngectomy”, “voice”, “speech”, “electrolarynx”, “esophageal”, “tracheoesophageal”, “acoustics”, “intelligibility”, “voice quality”, “quality of life” and their synonyms. The criteria for inclusion were that the written language was English, Dutch, German, Spanish or French. No filter for publication date was applied, and the search was performed in January 2016, with an update in December 2016.

All study types were included. Publications were included when at least two types of speech methods were compared. In these cases the same procedures for acoustic and perceptual evaluations are warranted for the different speaker groups within one study. Speaker groups had to be ≥ *n* = 7. There had to be a comparison of two or more speaker groups within the study. At least one of the primary outcome measurements had to be reported. Studies were graded according to the criteria of risk on bias described by the Cochrane Handbook for Systematic Reviews of Interventions, shown in the appendix, i.e., A = low risk of bias, B = unclear risk of bias, and C = high risk of bias [25, 26]. Level A and level B rated studies were included. Articles were excluded when they mainly reported on device research, primary or secondary voice prosthesis placement, adverse effects, or on pulmonary rehabilitation. Articles only reporting on secondary outcomes or rated as a Level C article were excluded. Reference lists were checked to collect more data. Studies which were rated with low risk of bias (level A) were indicated as best evidence available. These, level A rated studies are highlighted in the result section, as well as the significant outcomes reported by the included studies (level A and B). Only predefined outcome parameters were taken into account. Outliers were excluded in the overall “Results” section and are elaborated on further in the “Discussion” section. Outcomes of included studies were analyzed and, where possible, pooled. Data were tabulated and graphically represented in violin plots.

## Results

**Fig. 1:**
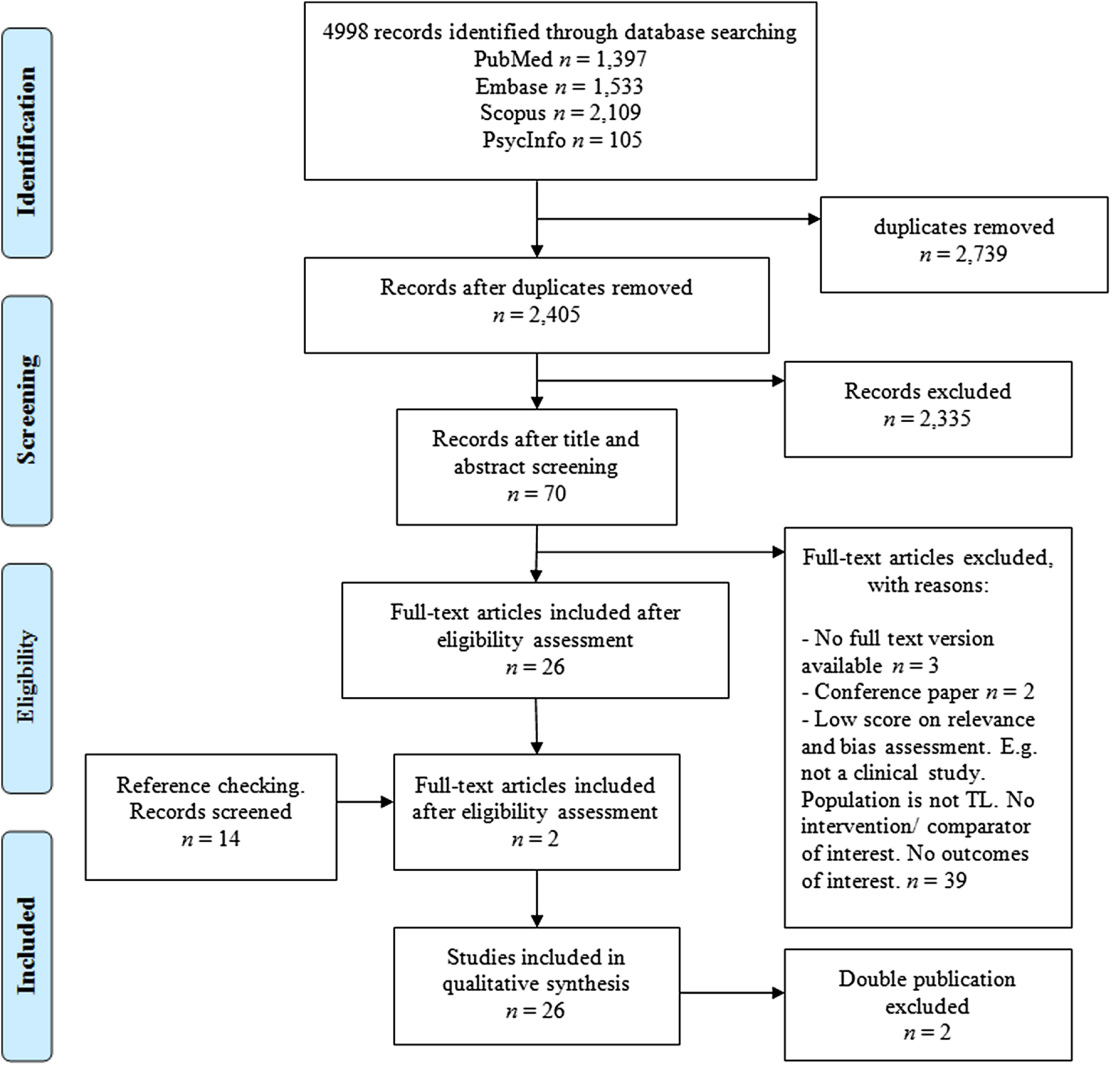
Flow diagram of study inclusion

The search and selection process is visualized in Fig. 1. The first and second author screened 50 out of 2405 papers on title and abstract to meet the selection criteria for inclusion. The first author performed the remaining screening of titles and abstracts. Seventy papers were evaluated in full for relevance and validity by the first and second author. References of the articles that were retrieved in full were screened, which resulted in two additional articles. The first and second author both performed a critical appraisal of the design of the studies. The third author evaluated all non-English articles. A decision on the inclusion of the articles was made in consensus of the three raters.

The definitive selection included 28 publications. There were two papers that discussed the same study, but were written in two different languages [27, 39]. We included the English version [27]. Furthermore, there were two publications of the same author published in 2013 and 2015, the 2015 paper containing additional speakers and evaluations to the 2013 paper [40, 41]. Therefore, we have chosen to only report on the 2015 paper in this systematic review [41]. This left 26 papers for further evaluation (Table 2).

**Table 2:** Overview of specifications of the 26 included studies and their risk of bias assessment

References	Total, *N*	ES, *n*	TES, *n*	ELS, *n*	H, *n*	Incl criteria	Gender	Age	Tumor location	Tumor stage	Treatment details	Time post TL	Acoustic outcomes	Perceptual outcomes	PROs	Clear tables and figures	Reliability checks	Blinding	< 30% loss-	Risk of Bias [26]
Arias et al. [27]	60	20	20	–	20	N	Y	Y	N	N	Y	N	Y	N	N	Y	N	–	Y	B
Bellandese et al. [32]	26	9	7	–	10	Y	Y	Y	N	N	Y	Y	Y	N	N	Y	Y	–	N	B
Blood [33]	30	10	10	–	10	N	N	Y	N	N	N	N	Y	N	N	Y	N	–	Y	B
Carello and Magnano [34]	14	7	7	–	–	N	Y	Y	N	N	N	N	Y	N	N	Y	N	–	Y	B
**Crosetti et al**. [42]	24	–	12	–	12	Y	Y	Y	Y	N	Y	Y	N	Y	N	Y	Y	Y	Y	**A**
De Maddalena et al. [43]	88	28	53	7	–	Y	Y	Y	N	N	N	N	N	Y	N	N	N	N	Y	B
DeBruyne et al. [44]	24	12	12	–	–	N	Y	Y	N	N	N	N	Y	N	N	N	N	–	Y	B
Deore et al. [29]	60	–	30	–	30	N	Y	N	Y	N	Y	N	Y	N	N	Y	N	–	Y	B
**Eadie et al**. [41]	36	2	23	11	–	Y	Y	Y	N	N	Y	Y	N	Y	Y	Y	Y	Y	Y	**A**
Evans et al. [45]	49	12	26	11	–	Y	Y	Y	N	N	Y	Y	N	N	Y	Y	–	N	Y	B
Finizia et al. [30]	22	–	12	–	10	Y	Y	Y	Y	Y	N	Y	Y	Y	N	N	Y	–	Y	B
Granda et al. [31]	18	10	8	–	–	Y	Y	Y	N	N	Y	Y	Y	Y	Y	Y	N	–	Y	B
Kinishi and Amatsu [35]	35	5	20	–	10	N	Y	Y	Y	Y	N	Y	Y	N	N	Y	N	–	Y	B
Law et al. [46]	34	7	13	14	–	Y	Y	Y	N	N	N	Y	N	Y	Y	Y	Y	N	Y	B
Maccallum et al. [47]	20	10	–	–	10	N	Y	Y	N	N	N	N	Y	N	N	Y	N	–	Y	B
Merol et al. [36]	59	30	29	–	–	Y	Y	Y	N	N	Y	N	Y	N	N	N	–	–	Y	B
Miralles and Cervera [48]	40	10	20	–	10	Y	Y	Y	N	N	N	Y	N	Y	N	N	N	N	Y	B
Mourkabel et al. [49]	75	15	42	18	–	Y	Y	Y	N	Y	Y	Y	N	N	Y	Y	–	–	Y	B
Ng et al. [50]	42	15	12	15	–	Y	Y	Y	N	N	N	N	N	Y	N	N	Y	N	Y	B
Robbins et al. [37]	45	15	15	–	15	N	Y	Y	N	N	Y	N	Y	N	N	Y	N	–	Y	B
Rosso et al. [51]	48	13	20	15	–	Y	Y	Y	N	N	N	N	N	N	Y	Y	–	–	Y	B
Salturk et al. [52]	96	24	57	15	–	Y	N	Y	N	N	N	N	N	N	Y	Y	N	–	Y	B
**Shim et al**. [28]	40	20	–	–	20	Y	Y	Y	Y	N	Y	Y	Y	N	N	Y	Y	–	Y	**A**
Siric et al. [38]	20	10	10	–	–	N	Y	Y	N	N	Y	N	Y	N	N	Y	N	–	Y	B
Tiple et al. [53]	49	17	14	18	–	Y	Y	Y	N	N	N	Y	N	N	Y	N	N	N	Y	B
Williams and Watson [54]	43	12	10	11	10	N	N	Y	N	N	N	Y	N	Y	N	N	Y	N	Y	B

Answers are indicated by: *Y* yes, the study fulfilled the criterion; *N* no, the study did not fulfill the criterion; – insufficient information provided or the study did not address the particular outcome. In bold the publications rated with level A (low risk of bias) in the risk of bias analysis

*ES* esophageal speakers, *TES* tracheoesophageal speakers, *H* healthy speakers, *TL* total laryngectomy, *PROs* patient-reported outcomes

In Table 2 details of the selected studies are provided. The scope of the research, the number of included participants and risk of bias rating is shown. In total, only three of the 26 studies (12%) reached level A (low risk of bias), shown in bold [28, 41, 42]. The remaining articles reached level B (unclear risk of bias).

A total of 1097 participants are included in the studies, only the groups of interest are taken into account. Groups of interest were ES (*n* = 313), TES (*n* = 482), ELS (*n* = 135), and a control group of healthy, i.e., laryngeal speakers (*n* = 167). Six studies only included male participants [29–31, 44, 45, 48]. One study only included female participants [32]. When gender is reported in studies, control groups are matched on or comparable to the sex of the TL groups. Fourteen studies [27–38, 44, 47] reported acoustic outcomes; nine studies [30, 31, 41–43, 46, 48, 50, 54] reported perceptual outcomes; and eight studies [31, 37, 41, 45, 46, 49, 51, 53] reported PROs. Four studies [30, 31, 41, 46] reported a combination of outcome measures.

Ten studies [27, 29, 33–35, 37, 38, 44, 47, 54] did not mention any inclusion criteria. Eleven studies [27, 29, 32–35, 43, 47, 48, 51, 54] failed to describe their method of selection and recruitment of participants. Two studies [30, 36] provided a detailed description of the selection process. The remaining articles [28, 37, 41, 44–46, 49, 50] mentioned the selection process briefly. Most patients were recruited via a clinical setting or via support groups.

Treatment details of the included groups were only provided in about half of the studies [27–29, 31, 32, 36–38, 41, 42, 45, 49]. This variable was indicated as present when treatment details were provided. Nevertheless, surgical details, e.g., use of flaps during the surgery were not provided in any of the included studies.

### Acoustic outcomes

In Table 3 acoustic outcomes for the included studies that reported on the primary (e.g., *F*_0_, HNR, MPT) and secondary outcomes (e.g., jitter, shimmer, intensity, spectral tilt) are presented. Comparative results for the different speaker groups are shown. None of the studies performed acoustical analysis on ELS, therefore ELS is not discussed in this section.

**Table 3:** Comparative acoustic outcomes for speaker groups

	*F*_0_ vowel	*F*_0_ speech	HNR	MPT	Jitter	Shimmer	Intensity	Spectral tilt
ES > TES	Granda et al. [31] Merol et al. [36]	–	–	–	–	Siric et al. [38]	–	–
ES > H	Shim et al. [28]	–	–		–	–	–	–
TES > ES	Arias et al. [27]* Bellandese et al. [32]* Blood [33]* Carello and Magnano [34] Kinishi and Amatsu [35] Robbins et al. [37] Siric et al. [38]*	Bellandese et al. [32] Robbins et al. [37] Blood [33]*	Arias et al. [27] Bellandese et al. [32] Carello and Magnano [34] Granda et al. [31] Siric et al. [38]	Carello and Magnano [34] Debruyne et al. [44] Granda et al. [31] Merol et al. [36] Robbins et al. [37] Siric et al. [38]*	Arias et al. [27] Carello and Magnano [34] Kinishi and Amatsu [35] Robbins et al. [37] Siric et al. [38]	Arias et al. [27] Robbins et al. [37]	Blood [33] Granda et al. [31] Robbins et al. [37] Siric et al. [38]*	DeBruyne et al. [44]
TES > H	–	–	–	–	–	–	–	–
H > ES	Arias et al. [27] Bellandese et al. [32] Blood [33] Kinishi and Amatsu [35]	Bellandese et al. [32] Blood [33]* Robbins et al. [37]	Arias et al. [27]* Bellandese et al. [32] Maccallum et al. [47]* **Shim et al.** [28]*	Robbins et al. [37]	Arias et al. [27]* Kinishi and Amatsu [35] Maccallum et al. [47]* Robbins et al. [37] **Shim et al.** [28]*	Arias et al. [27]* Maccallum et al. [47]* Robbins et al. [37] **Shim et al.** [28]*	Blood [33]* Robbins et al. [37]	**Shim et al.** [28]*
H > TES	Arias et al. [27]* Bellandese et al. [32] Blood [33] Deore et al. [29]* Finizia et al. [30] Kinishi and Amatsu [35] Robbins et al. [37]	Bellandese et al. [32] Blood. [33]* Finizia et al. [30] Robbins et al. [37]	Arias et al. [27]* Bellandese et al. [32] Deore et al. [29]*	Deore et al. [29]* Finizia et al. [30] Robbins et al. [37]	Arias et al. [27]* Deore et al. [29]* Kinishi and Amatsu [35] Robbins et al. [37]	Arias et al. [27] Deore et al. [29] Robbins et al. [37]	Blood [33] Robbins et al. [37]	–

> Indicating a better mean group outcome. Papers reporting significance are indicated with an *(*p* ≤ .05). Studies presented in bold had a level A risk of bias

*ES* esophageal speakers, *TES* tracheoesophageal speakers, *H* healthy speakers, *F*_*0*_ fundamental frequency, *HNR* harmonics to noise ratio, *MPT* maximum phonation time

#### Fundamental frequency

**Fig. 2:**
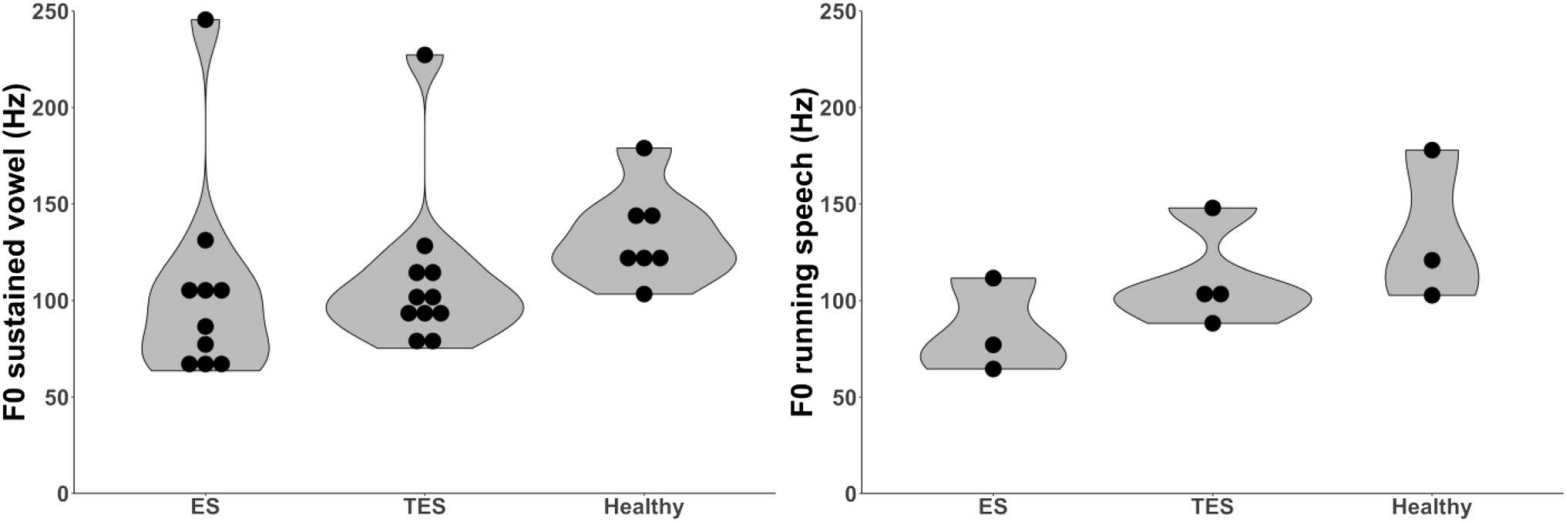
Left violin plot displaying the distribution of the mean *F*_0_ outcomes for sustained vowels in 12 studies [27–38] ES *n* = 136, TES *n* = 168, H *n* = 115. Right violin plot displaying the distribution of the mean *F*_0_ outcomes for running speech in four studies [30, 32, 33, 37]. ES *n* = 34, TES *n* = 44, H *n* = 35. *ES* esophageal speakers, *TES* tracheoesophageal speakers, *ELS* electrolarynx speakers, *H* healthy speakers, *F*_*0*_ fundamental frequency, *Hz* hertz

Thirteen papers [27–38, 44] (*n* = 443) reported fundamental frequency (*F*_0_) outcomes, including the level A categorized study of Shim et al. [28]. Measurements are presented for evaluations in sustained vowels and in running speech (Table 3; Fig. 2). No distinction between male and female speakers is made. Most studies did not make this distinction since the sound source, the PE-segment, is similar in both groups. Not all *F*_0_ outcomes could be taken in account because in some studies the reporting was only range of *F*_0_ or in boxplots [30, 44]. Higher *F*_0_ values are designated as better [1]. The total range of *F*_0_ values for all groups of speakers in vowels and running speech is 64–179 Hz, which is reported in twelve studies [27–38]. The mean *F*_0_ value of 227 Hz for TES [38] and the mean *F*_0_ value of 246 Hz for ES [31] are considered outliers and were therefore excluded. The level A rated study of Shim et al. [28] showed non-significant higher mean *F*_0_ values for ES compared to healthy speakers, resp. 131 Hz and 124 Hz. Higher mean *F*_0_ values are found for healthy speakers compared to ES and TES (*N* = 7) [27, 29, 30, 32, 33, 35, 37]. In two studies this difference was significant [27, 33]. For the speech rehabilitation methods higher *F*_0_ values are seen in the group of TES as compared to ES (*N* = 7) [27, 32–35, 37, 38]. In four studies this difference was significant [27, 32, 33, 38].

#### Harmonics to noise ratio

**Fig. 3:**
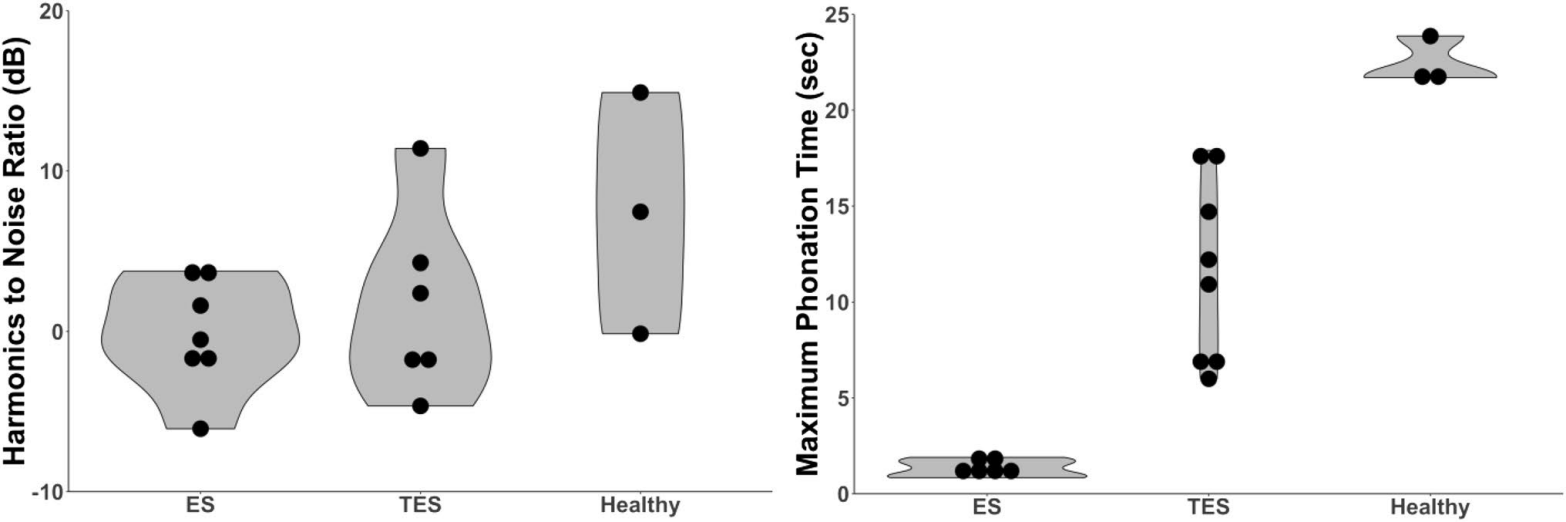
Left violin plot displaying the distribution of the mean HNR outcomes in eight studies [27–29, 31, 32, 34, 38, 47]. ES *n* = 86, TES *n* = 82, H *n* = 90. Right violin plot displaying the distribution of the mean MPT outcomes in eight studies [29–31, 34, 36–38, 44]. ES *n* = 84, TES *n* = 123, H *n* = 55. *ES* esophageal speakers, *TES* tracheoesophageal speakers, *H* Healthy speaker, *ELS* electrolarynx speakers, *HNR* harmonics to noise ratio, *MPT* maximum phonation time

Eight studies [27–29, 31, 32, 34, 38, 47] *(n* = 240) included Harmonics to Noise Ratio (HNR) outcomes, including one level A study of Shim et al. [28], and seven level B studies [27, 29, 31, 32, 34, 38, 47]. Two studies measured Noise to Harmonics Ratio, which was recalculated to HNR [28, 34]. Higher HNR is reflecting better voice quality. The level A rated study of Shim et al. [28] showed better HNR outcomes for healthy speakers compared to ES. Healthy speech was rated as superior to substitute speech in five studies [27–29, 32, 47]. Comparison between ES and TES showed superior values for TES (Table 3; *N* = 5) [27, 31, 32, 34, 38]. However, none of the studies comparing ES and TES found a significant difference between these substitute voices. This is reflected in the violin plots in Fig. 3, depicting the comparable HNR outcomes for TES and ES.

#### Maximum phonation time

Eight level B rated studies [29–31, 34, 36–38, 44] (*n* = 262) evaluated Maximum Phonation Time (MPT) by phonation of a vowel /a/ or /i/ (Table 3; Fig. 3). Longer MPT is indicated as being better. Range of MPT values for all groups of speakers is 0.84–23.87 s (*N* = 8) [29–31, 34, 36–38, 44]. In the group of healthy speakers values for MPT are highest [29, 30, 37]. A significant longer MPT for healthy speakers is found in comparison with TES [29]. Within the comparison of the speech rehabilitation groups six studies [31, 34, 36–38, 44] found that TES has a longer MPT than ES, significant in one study [38].

#### Jitter

**Fig. 4:**
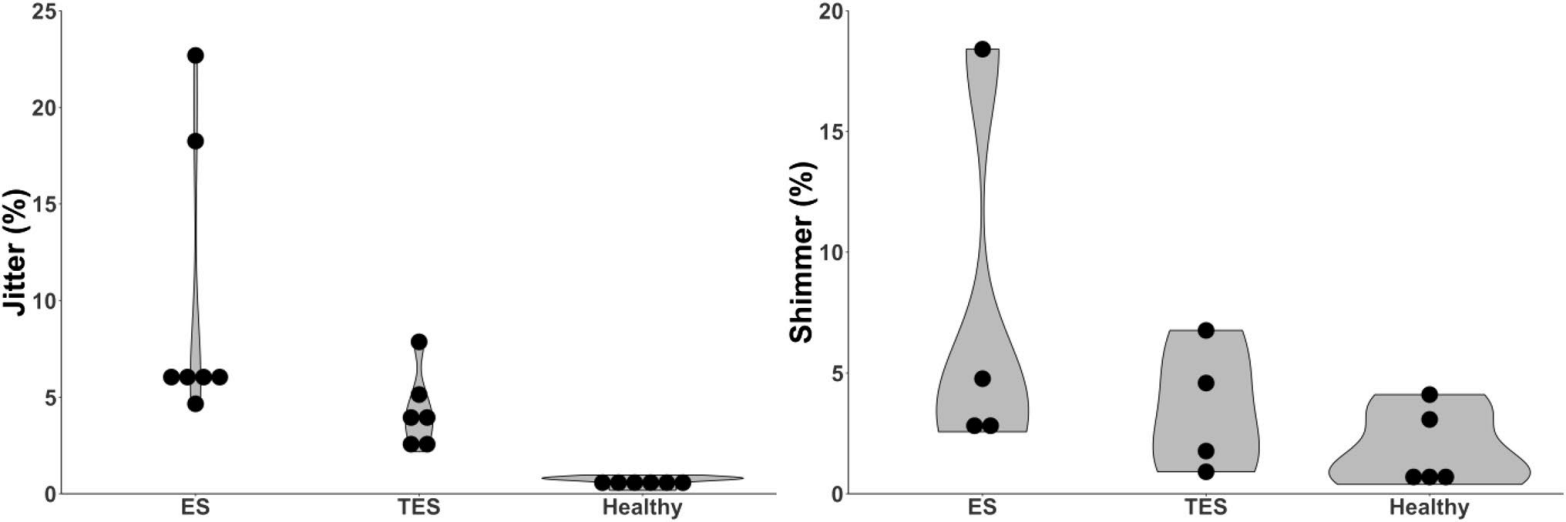
Left violin plot displaying the distribution of the mean jitter outcomes of eight studies [27–29, 34, 35, 37, 38, 47] ES *n* = 87, TES *n* = 102, H *n* = 105. Right violin plot displaying the distribution of the mean shimmer outcomes of five studies ES *n* = 65, TES *n* = 65, H *n* = 95 [27–29, 37, 47]. *ES* esophageal speakers, *TES* tracheoesophageal speakers, *H* healthy speaker, *ELS* electrolarynx speakers, *HNR* harmonics to noise ratio, *MPT* maximum phonation time

Comparison of jitter values between groups were made in eight studies [27–29, 34, 35, 37, 38, 47] (*n* = 294). One study was categorized as level A [28] and seven studies as level B [27, 29, 34, 35, 37, 38, 47]. Low jitter value is related to better voice quality [9, 20]. Lowest values for jitter are found in the group of healthy speakers (*N* = 6 [27–29, 35, 37, 47], Fig. 4). A significant difference between healthy speakers and the groups of substitute voice speakers is found in four studies, one of which is listed a level A study [28], and three as level B [27, 29, 47] (Table 3; Fig. 4). For the groups of substitute voice speakers lower jitter values were found for TES compared to ES, but these outcomes were not significant (*N* = 5 [27, 34, 35, 37, 38], Table 3).

#### Shimmer

Six studies [27–29, 37, 38, 47] (*n* = 245) reported on shimmer values, of which one study was categorized as level A [28], five as level B [27, 29, 37, 38, 47]. Outcomes for shimmer in percentage were compared. For two studies [37, 38], shimmer outcomes were presented in shimmer decibel. For this systematic review, these data were recalculated to percentages [37, 38]. A low shimmer value is related to a better voice quality [9, 20]. In the level A study of Shim et al. [28] significantly better shimmer outcomes are reported for healthy speakers compared to TES (Table 3). Shimmer values for all groups ranged from 0.4 to 18.4%. The value of 53.6% shimmer for ES, is considered to be an outlier [47]. It seems that based on shimmer, healthy speech can be rated as superior compared to ES and TES (*N* = 5 [27–29, 37, 47], Table 3, Fig. 4). No definite trend is seen between the speech rehabilitation methods ES and TES (*N* = 3, Table 3) [27, 37, 38].

#### Intensity

Four level B categorized studies [31, 33, 37, 38] (*n* = 113) reported intensity scores (Table 3). Intensity in decibel is not an absolute value, and therefore comparing the mean outcome is irrelevant. A higher intensity is indicated as being better [1]. Highest intensity scores are found for healthy speakers [33, 37]. Between substitute voices four studies [31, 33, 37, 38] found higher intensity scores for TES than for ES. In only one of these studies the higher intensity for TES compared to ES was significant [38].

#### Spectral tilt

Two studies [28, 44] (*n* = 64) calculated the ratio between the energy above 4 kHz and the energy in the lower frequencies. One study was categorized as level A [28], the other as level B [44]. A larger ratio is correlating with better voice quality [55]. The level A rated study of Shim et al. [28] reported a larger ratio in healthy speakers than in ES. In the other study [44] the spectral tilt ratio was found to be larger for TES than for ES. It is not possible to draw overall conclusion from this due to the small number of studies reporting this outcome measure.

### Perceptual outcomes

In Table 4, comparative perceptual results for the different speaker groups are shown. Studies that reported on the primary outcomes “voice quality” and “intelligibility” are presented. Initially formulated outcome variables, which were not reported in the included studies, cannot be discussed. This concerns the percentage of voicedness, “Grade Roughness Breathiness Asthenia Strain scale assessment” (GRBAS), unattended additive noise, fluency and voicing.

**Table 4:** Comparative perceptual and patient-reported outcomes for speaker groups

	Perceptual voice quality	Perceptual intelligibility	PROs VHI	PROs V-RQOL
ES > TES	–	–	Salturk et al. [52]* Tiple et al. [53]	–
ES > ELS	Ng et al. [50]	Williams and Watson [54]*	Salturk et al. [52]* Tiple et al. [53]	Moukarbel et al. [49]
ES > H	–	–	–	–
TES > ES	Law et al. [46] Ng et al. [50] Williams and Watson [54]*	Law et al. [46] Ng et al. [50] Williams and Watson [54]*	–	Moukarbel et al. [49]
TES > ELS	Eadie et al. [41]* Ng et al. [50] Miralles and Cervera [48] Williams and Watson [54]*	Eadie et al. [41]* Ng et al. [50] Williams and Watson [54]*	Eadie et al. [41]	Moukarbel et al. [49]*
TES > H	–	–	–	–
ELS > ES	Law et al. [46]	Law et al. [46] Ng et al. [50]	–	–
ELS > TES	Law et al. [46]	Law et al. [46]	Tiple et al. [53]	–
ELS > H	–	–	–	–
H > ES	Williams and Watson [54]*	Williams and Watson [54]*	–	–
H > TES	Finizia et al. [30] Williams and Watson [54]*	Finizia et al. [30] Williams and Watson [54]* Crosetti [42]*	–	–
H > ELS	Williams and Watson [54]*	Williams and Watson [54]*	–	–

> Indicating a better mean group outcome. Papers reporting significance are indicated with an *(*p* ≤ .05). Studies presented in bold had a level A risk of bias

*ES* esophageal speakers, *TES* tracheoesophageal speakers, *ELS* electrolarynx speakers, *H* healthy speakers, *VHI* voice handicap index, *V-RQOL* voice-related quality of life

#### Voice quality

Voice quality was perceptually evaluated in five studies [30, 41, 46, 50, 54] (*n* = 177). One of these studies was categorized as level A [41], four level B [30, 46, 50, 54]. Across these studies, different evaluation methods were used. In the level A study by Eadie et al. [41] speech acceptability ratings were obtained for ES, TES and ELS measured by a visual analog scale (VAS). The audio recordings were evaluated by 48 listeners, and speakers judged their own speech acceptability. In another study [46] the evaluators were instructed to rate the severity of the speech impairment on an 11-point scale with equal-appearing interval, from no speech impairment to a severe speech impairment. This study made a distinction between younger and older listeners, and in the present review the results for both groups of listeners were pooled [46]. One study [30] performed perceptual evaluation of TES compared to healthy speakers on a VAS 0-100. Another study [50] used a one to seven equal-interval scale for rating. A score of one indicated severe hoarseness, while a score of seven indicated a clear voice quality. A scale of one to seven was also used in another study [54], in which evaluators rated videotaped speaking fragments of ES, TES and ELS for voice quality [54].

The level A rated study of Eadie et al. [41] found that TES are rated with a significant more acceptable voice quality compared to ELS (Table 4). When speakers had to judge their own voice recordings no differences were found in speech acceptability between TES and ELS [41]. Two other studies [48, 50] also found better voice quality outcomes for TES compared to ELS, though these differences were non-significant [48, 50]. Three studies [46, 50, 54] found a better voice quality for TES compared to ES, which was significant in one of these [54]. In one study [46], ELS was rated as having a better voice quality compared to ES and TES. Another study indicated ES as having a better voice quality compared to ELS [50]. When a comparison between substitute voice speakers and healthy speakers is made, the group of healthy speakers is rated to have the best voice quality [30, 54]. One study underlined this with significance [54].

#### Intelligibility

In eight studies [30, 41–43, 46, 48, 50, 54] (*n* = 329) intelligibility was assessed, and two of these were level A studies [41, 42], and six level B studies [30, 43, 46, 48, 50, 54]. Different methods were used to measure intelligibility outcomes. Two studies [50, 54] used a self-developed seven-equal interval scale, while one study [30] used a VAS of 0-100. The VAS and interval scales were used to rate the speakers’ intelligibility. Two studies [41, 46] used the Sentence Intelligibility Test (SIT) to perform a per protocol analysis. Other studies developed their own assessment procedure. One study [43] evaluated TES with a group including ES as well as ELS under different conditions. Outcomes are presented with the percentage of correct heard words [43]. Another study [42] performed a telephone listening task where words and sentences of TES and healthy speakers were transcribed. One more study [48] evaluated audio samples on intelligibility in ES and TES by phoneme confusion matrices. In this study no overall intelligibility scores were given [48].

Comparative group outcomes are shown in Table 4. For the speech rehabilitation methods TES is mostly rated to have a better intelligibility compared to ES and ELS. In two studies [41, 54] including the level A rated study of Eadie et al. [41], this is confirmed with significance [41, 54]. In one study, ES is rated to have a significantly better intelligibility compared to ELS [54]. The intelligibility of healthy speakers is rated as superior compared to the speech rehabilitation methods. Two studies [42, 54], including the level A rated publication of Crosetti et al. [42], indicated that this difference was significant.

In one study [43], a comparison was made between the group of TES and a group of ES combined with ELS, and therefore, this study is not presented in Table 4. TES scored significant better on intelligibility when communicating with strangers in a situation where there was no face-to-face contact [43]. One study [48] found that for TES, fricative consonants had the highest number of confusions. Whereas in ES, nasals caused the highest number of confusions [48]. There was no significant difference with regards to intelligibility found between ES and TES [48].

### Patient-reported outcome

In Table 4 patient-reported outcomes (PROs) for the included studies are presented. Comparative results for the different speaker groups are shown. Predefined outcome variables, which were formulated in the research question but not reported in the included studies, will not be discussed. This concerns the EORTC QLQ-H&N35, and EORTCQLQ-C30. None of the studies compared PROs on voice quality of healthy speakers with alaryngeal speakers. Therefore, only comparative outcomes of the speech rehabilitation methods can be shown.

#### Voice Handicap Index

The Voice Handicap Index (VHI) was used in six studies [31, 41, 45, 51–53] (*n* = 296). Four level B studies [31, 45, 51, 53] used the full version of the VHI. Two studies, including the level A rated study of Eadie et al. [41], used the ten item version of the VHI [41, 52]. In two studies [31, 51] no mean outcomes per speaker group were reported, only number of speakers per severity level were reported. One study [45] compared TES to a group of speakers using other, non-surgical voice restoration methods (EL, ES, mouthing, and writing). In the present review, this comparison of TES with a heterogeneous group of speakers was not taken into account (TES *n* = 35 compared to non-surgical voice restoration *n* = 27) [45].

One study [52], reports significant better vocal functioning for ES compared to the groups of TES and ELS [52] (Table 4). In the level A rated study of Eadie et al. [41] no differences were found between TES and ELS. Although the scores of TES were slightly better, these differences were not significant (Table 4) [41]. Additionally, three level B rated studies [31, 51, 53] did not find any significant differences between groups. In one study [31] ES and TES both reported a slight or moderate perceived voice handicap. In another study [51] ES, TES and ELS reported a moderately perceived voice handicap, and no group differences were found. Furthermore, the study of Tiple et al. [53], did not find any significant differences between ES, TES and ELS. According to this study, however, having no communication method available at all leads to a significantly worse VHI score compared to having ES as communication option [53].

#### Voice-related quality of life

One level B rated study [49] (*n* = 75) used the Voice-Related Quality of Life (V-RQOL) within the groups of ES, TES and ELS [49]. ES reported a better V-RQOL compared to ELS. TES reported a better V-RQOL than ES and ELS. The V-RQOL of TES was significantly better compared to ELS [49].

### Summary of results

In Table 5 a summary of the significant differences between the speech rehabilitation is provided. Comparative studies of the three rehabilitation methods themselves show that TES is rated as superior to ES for the acoustic outcome measures *F*_0_, MPT and intensity [27, 32, 33, 38], whereas no acoustic data are available for ELS in the included studies. According to the applied perceptual evaluations, TES is rated as superior to ES and ELS, with regards to both voice quality and intelligibility. ES is superior to ELS for the perceptual outcome measure intelligibility. In PRO studies, none of the speech rehabilitation methods showed evidently better outcomes. One study reported significant better outcomes for TES compared to ES [49], but another study showed the opposite [52]. A level A rated study reports a similarly moderate degree of perceived voice handicap in TES and ELS [41].

**Table 5:** Summary of Tables 4 and 5, studies that found a significant difference between speech methods per outcome measure

	TES > ES	TES > ELS	ES > ELS	ES > TES
Fundamental frequency	Arias et al. [27] Bellandese et al. [32] Blood [33] Siric et al. [38]	–	–	–
MPT	Siric et al. [38]	–	–	–
Intensity	Siric et al. [38]	–	–	–
Perceptual voice quality	Williams and Watson [54]	Eadie et al. [41] Williams and Watson [54]	–	–
Perceptual intelligibility	Williams and Watson [54]	Eadie et al. [41] Williams and Watson [54]	Williams and Watson [54]	–
PROs	–	Moukarbel [49]	Salturk et al. [52]	Salturk et al. [52]

> Indicating a better mean group outcome. Level of significance was held at p ≤ .05. Studies presented in bold had a level A risk of bias

*ES* esophageal speakers, *TES* tracheoesophageal speakers, *ELS* electrolarynx speakers, *MPT* maximum phonation time, *V-RQOL* voice-related quality of life

## Discussion

This systematic review underlines that the three main TL-speech rehabilitation methods are acoustically and perceptually deviant from healthy speech. In PROs no comparison is made between the substitute speech rehabilitation groups and healthy speakers. Significantly better outcomes are reported for TES compared to ES for the acoustic parameters, fundamental frequency, maximum phonation time and intensity. Perceptually, TES is rated with a significantly better voice quality and intelligibility than ES and ELS. None of the speech rehabilitation groups reported evidently better outcomes in patient-reported outcomes.

These outcomes need to be interpreted with caution. Only three of the 26 included studies are rated with a low risk of bias (level A). Most outcomes, thus, stem from level B rated studies. The included studies contain small numbers of patients, and inferential statistics is not always performed. In most studies the methodology of the acoustic measurements was not specified, leading to possibly incorrect outcomes. We found several extreme outliers in *F*_0_ and shimmer, that we had to exclude because of this [31, 38, 47]. Difficulties in reliable measuring intensity values are acknowledged, no absolute values are reported but only outcomes within studies.

This systematic review shows once more that there is an urge for standardized measurement tools for evaluations of substitute voice speakers. Auditory-perceptual evaluations are often considered as being the gold standard for voice and speech evaluation. However, the great dispersion between raters has to be acknowledged. Researchers have proposed rating tools for standardized evaluations [7, 16, 18]. Nevertheless, these are not yet generally adopted. An interesting new approach is the development of automatic assessment tools, which are designed to provide objective outcomes, with some promising results recently being reported [56, 57]. Even though not all present automatic assessment tools seem suitable for analyzing substitute voices, in our opinion this is the most promising way to obtain objective voice outcomes.

The number of PRO studies that could be included in this review is limited. The EORTC QLQ-H&N35 and EORTCQLQ-C30 were defined as relevant outcome measure but not reported in the included studies. We did not find studies which specifically report the outcomes on the speech domain of these questionnaires for the different speaker groups. The VHI and V-RQOL are generally applied to evaluate vocal functioning after TL. The Communication and Participation Item Bank (CPIB) is a recently developed questionnaire [41], which is why it was not initially defined as an outcome of interest. However, the level A rated study of Eadie et al. [41] showed strong correlations between the VHI-10 and CPIB short form scores. In this study, the speech rehabilitation groups were asked to judge their own voice quality and intelligibility. These outcomes also strongly correlated with the CPIB short form scores. Therefore, the CPIB short form can be seen as a useful tool to obtain patients’ opinion on vocal functioning which fits within the widely applied International Classification of Functioning (ICF) [58].

TES has favorable outcomes on the acoustic variables *F*_0_, MPT and intensity compared to ES. Both speech methods are generated within the same sound source, the PE-segment. The most likely explanation for the more favorable acoustic voice outcomes in TES is that this type of speech is pulmonary driven. It is feasible that with the pulmonary airflow, the tidal volume (roughly 5–600 ml) of TES, a more stable and better controlled airflow is created. The higher pressure could lead to controlled hypertonicity or a movement of the PE-segment to a more cranial position. This can be an explanation for the higher *F*_0_ values which are found in TES. For ES only a minimal volume of air is available, about 60–80 ml, which is roughly 2% of the lung capacity, and controlling the pressure is not really possible [1]. This limited airflow and volume lead to a shorter phonation time for ES and, presumably, to a lower *F*_0_ and lower intensity.

No limitation in publication date was applied. Several studies published in the 1980s and 1990s were included. During the 1980s ES was known as the gold standard for speech rehabilitation, and TES was just introduced. It is likely that ES was educated fairly well in this decade. Esophageal speakers may have achieved more satisfactory outcomes in the earlier publication period than present.

We assume that the result of the speech rehabilitation efforts plays a role in self-reported outcomes. Especially ES patients often require a prolonged and intensive rehabilitation period and success is not guaranteed. Therefore, ES speakers could be more satisfied and proud of their accomplishments than TES speakers, who acquire their speech more rapidly.

For the studies included in this systematic review, it is very likely that recruitment bias exists. In most studies, recruiting and selection of participants is not described. For the acoustic and perceptual outcomes, it can be assumed that only speakers with a fairly good level of speech were included. Some authors report this bias by mentioning that they only included excellent speakers [27, 32, 33, 38, 50]. One study [52] reported that all patients in the group of ELS failed to achieve intelligible ES and five failed in TES [52]. Additionally, studies reported that they had to exclude participants because of lack of speech performance or had to exclude audio files from acoustic analysis due to lack of periodicity [28, 44].

Obviously, there are more aspects influencing the acoustic, perceptual and PROs of speech rehabilitation after TL. In Table 6 several of these aspects related to the speech rehabilitation methods are listed. Valuable information that can explain functional outcomes after TL is missing in the reported studies. Most studies do not mention treatment details or time since TL. Furthermore, information about offered speech rehabilitation methods and aggregate practice time with the speech language pathologist is lacking. Knowledge of caretakers and differences in health care and insurance systems play a role in the speech rehabilitation options that can be offered. Also, patients’ personal factors should be taken into account when offering speech rehabilitation. Medical problems such as neurological disorders, causing a lack of dexterity or trainability, can hamper any rehabilitation technique and influence the choice. Societal participation, which includes family life and employment status also plays a role in patients’ preference for a speech rehabilitation method.

**Table 6:** Aspects of the three speech rehabilitation methods with regards to required equipment, costs and dependence on healthcare system [1, 50, 53, 59–62]

	Esophageal speech (ES)	Tracheoesophageal speech (TES)	Electrolarynx speech (ELS)
Mechanical or prosthetic device required	No	Yes	Yes
Hand occupied during voicing	No	Yes/no, some patients are able to use an automatic speaking valve	Yes
Dependence on speech language pathologist (SLP)	Yes, nowadays fewer SLP’s have knowledge of providing ES therapy	Yes, knowledge of voice prosthesis equipment and TES rehabilitation is required	Yes
Duration of the therapeutic process to functional speech	Training time, mostly concerns several months	Useful speech is mostly achieved within five training sessions	Useful speech is mostly achieved within five training sessions
Financial implications	No material costs. More therapeutic costs during often prolonged training period	Material costs, higher than ES and ELS. Potential reimbursement issues. Lower therapeutic costs that ES, comparable to ELS	Material costs, lower than TES. Lower therapeutic costs that ES, comparable to TES
Overall success achieving useable speech	Low success rate	High success rate	High success rate

Besides voice quality, physical capacity, emotional well-being and social functioning are also affecting general quality of life in TL patients [63, 64]. Poor general condition is negatively associated with successful voice rehabilitation [65]. Additionally, the extent of the surgery pays a role, e.g., in case of a pharyngolaryngectomy even more functional speech problems and reduced quality of life is reported [66].

## Conclusions

This systematic review consists of 26 studies reporting on multidimensional voice outcomes after total laryngectomy. Only three of these studies could be rated with a low risk of bias. This number is insufficient to draw firm conclusions. Most studies were rated with a unclear risk of bias because of flaws in patient selection and methodology.

For acoustic outcomes, tracheoesophageal speech (TES) seems to be more comparable to healthy speech. Significantly better outcomes for fundamental frequency, maximum phonation time and intensity are found in TES more than in ES. TES seems to be most pleasant and comprehensible in the perceptual evaluations, followed by esophageal speech. Speaking with an electrolarynx was found to be least pleasant and comprehensible. For the PROs, all speaker groups report a degree of voice handicap. However, none of the speech rehabilitation methods were clearly indicated as achieving more satisfactory outcomes in self-reported vocal functioning.

## Electronic supplementary material

Below is the link to the electronic supplementary material.

Supplementary material 1 (DOCX 14 KB)
