# Supplementary material for: Objective and subjective voice outcomes after total laryngectomy: a systematic review
Source: Eur Arch Otorhinolaryngol. 2017 Oct 31;275(1):11–26. doi: 10.1007/s00405-017-4790-6 (PMC5754416; doi:10.1007/s00405-017-4790-6)
Supplement: Supplementary file 1 — Supplementary material 1 (DOCX 14 KB) [file 405_2017_4790_MOESM1_ESM.docx]

**Appendix.** Grading System for assessing risk of bias according the Cochrane Handbook [[21](#_ENREF_21)].

| **Risk of bias** | **Interpretation** | **Within a study** | **Across studies** |
| --- | --- | --- | --- |
| A. Low risk of bias | Plausible bias unlikely to seriously alter the results. | Low risk of bias for all key domains. | Most information is from studies at low risk of bias. |
| B. Unclear risk of bias | Plausible bias that raises some doubt about the results. | Unclear risk of bias for one or more key domains. | Most information is from studies at low or unclear risk of bias. |
| C. High risk of bias | Plausible bias that seriously weakens confidence in the results. | High risk of bias for one or more key domains. | The proportion of information from studies at high risk of bias is sufficient to affect the interpretation of results. |
